# Wanted: opinionated neuroscientists

**DOI:** 10.1186/2042-1001-1-14

**Published:** 2011-10-06

**Authors:** Anna Webb, Peter Latham, Venkatesh Murthy

**Affiliations:** 1 BioMed Central Ltd, London, UK; 2 Gatsby Computational Neuroscience Unit, University College London, London, UK; 3 Department of Molecular and Cellular Biology, and Center for Brain Science, Harvard University, Cambridge, MA, USA

## 

*Neural Systems & Circuits *is embarking on a new venture: we want to gauge the opinion of the community on a range of issues, both technical and ethical, surrounding modern neuroscience. Issues such as the benefits (and pitfalls) of integrating theory and experiment, working with large datasets, and open science. We will be inviting opinion pieces from leaders in many different areas of theoretical and experimental neuroscience, but would like to expand beyond the 'usual suspects' to hear the thoughts of young scientists. We are particularly keen to receive opinion pieces from graduate students and postdocs. Perhaps a new hypothesis has arisen from attending a particularly interesting meeting, a paper that went relatively unnoticed when first published but has since been proven seminal, a study that has recently caught your attention, or maybe you have a prediction of where a particular field will be in 10 years? All of these, and more, will be considered.

Essays should be one or two pages long, and will be selected based on their content as well as style. This is a fantastic opportunity to have your voice heard in the community, and to participate in the wider debate on the future of your field.

For enquiries regarding this series of articles, please contact: editorial@neuralsystemsandcircuits.com, or anna.webb@biomedcentral.com.

To submit an article to *Neural Systems & Circuits*, please visit: http://www.neuralsystemsandcircuits.com/manuscript.

We look forward to receiving your submissions!

